# Porous membrane with high curvature, three-dimensional heat-resistance skeleton: a new and practical separator candidate for high safety lithium ion battery

**DOI:** 10.1038/srep08255

**Published:** 2015-02-05

**Authors:** Junli Shi, Yonggao Xia, Zhizhang Yuan, Huasheng Hu, Xianfeng Li, Huamin Zhang, Zhaoping Liu

**Affiliations:** 1 Ningbo Institute of Materials Technology Engineering (NIMTE), Chinese Academy of Sciences, Zhejiang 315201, P. R. China; 2 Division of energy storage, Dalian Institute of Chemical Physics, Chinese Academy of Sciences, Dalian 116023, China

## Abstract

Separators with high reliability and security are in urgent demand for the advancement of high performance lithium ion batteries. Here, we present a new and practical porous membrane with three-dimension (3D) heat-resistant skeleton and high curvature pore structure as a promising separator candidate to facilitate advances in battery safety and performances beyond those obtained from the conventional separators. The unique material properties combining with the well-developed structural characteristics enable the 3D porous skeleton to own several favorable properties, including superior thermal stability, good wettability with liquid electrolyte, high ion conductivity and internal short-circuit protection function, etc. which give rise to acceptable battery performances. Considering the simply and cost-effective preparation process, the porous membrane is deemed to be an interesting direction for the future lithium ion battery separator.

Nowadays, the ever-increasing demand for high-energy density greatly promotes the development of electrochemical energy storage devices. Reliable, safe and high performance lithium ion batteries are an important trend in the future^1^,^2^,^3^. However, a series of accidents on battery catching fire and explosion have been reported, which has became a crucial bottleneck for the further development of lithium ion batteries^4^. Among the major battery components, a separator is one of the most significant ones, which acts as a physical barrier to prevent contact between the electrodes, preserve the liquid electrolyte and to conduct the lithium ions during the cycling process^5^. Considering its main functions, the role of a separator in enhancing battery safety and output performances is critical and could not be underestimated^6^,^7^,^8^,^9^.

Polyolefin separators are the most commercially available ones in the consumer market. However, the security mechanism is usually deemed to be effective for small cells (<3 Ah) at low voltage (<12 V)^6^. For high power/capacity lithium ion batteries in the application of transportation or energy storage, this kind of separators show severe lack of capacity. During the charge/discharge process of batteries at high C-rate and under the condition of battery abuse, a lot of heat would be generated and released, leading to a dramatic increase in battery temperature. So separators are required to own enhanced thermal stability, to avoid the occurrence of short circuit, thermal runaway and even explosion caused by separator shrinkage and melt down at high temperature. However, the melting temperature of polyolefin materials is normally in the range between 135°C and 165°C, which is below the onset temperature of most conventional lithium-ion chemistries (commonly higher than 200°C)^6^. In addition, the poor wettability of polyolefin separators with conventional liquid electrolyte has also brought serious disadvantages in manufacturing costs and battery performances^8^. Therefore, separators with good thermal stability and wettability are in urgent need for the development of high performamce lithium ion batteries.

Recently, two approaches have been developed to overcome the drawbacks of polyolefin separators. One strategy is introducing the inorganic ceramics onto/into the separators^10^,^11^. The other is constructing the heat-resistant nonwoven separators^12^,^13^,^14^. Nevertheless, these strategies are confined with complex technology, and high preparation cost. Besides, although the nonwoven separators are reported to own superior thermal stability, which are usually prepared from thermal plastics, the excessively large and straight-through pores have been found to induce internal short circuit, self-discharge and liquid electrolyte leakage seriously in many reports^9^,^15^. The report highlights our effort to construct a new class of battery separator that features 3D heat-resistance skeleton to enhanced battery thermal safety and high curvature pore structure to play in internal short circuit protection function, to overcome the shortcomings of conventional separators.

Polyether imide (PEI) is one kind of engineering plastics with good chemical and thermal stability (heat deflection temperature >200°C), excellent electrical insulation performances and inherent flame retardancy. Meanwhile, the polar amide bonds and ether bonds in the polymer chains are expected to strengthen the interaction between the separator and the high polar liquid electrolyte, which implies great promising application as separator matrix for lithium ion batteries. Here, the PEI base porous skeleton was constructed and the functionality in lithium ion batteries was firstly demonstrated.

Another notable advantage of PEI on other thermal plastics, such as polyimide (PI) and polyester (PET), is that it is easily dissolved in the commonly used solvents under mild conditions. To achieve the desired porous skeleton, vapor induced phase inversion process was operated, which is prone to form uniformly distributed and highly symmetric sponge-like pore, and could further effectively overcome the critical problems of currently used porous membranes (straight pores, low porosity) and will be highly beneficial to improving their performances in lithium ion batteries. The material uniqueness and the competitive structure properties of the PEI based porous skeleton/membrane, in combination with its good electrolyte wettability, are expected to allow substantial improvement in ion transfer between electrodes and to provide a new separator opportunity for the development of high-performance batteries.

## Results

**Figure 1:**
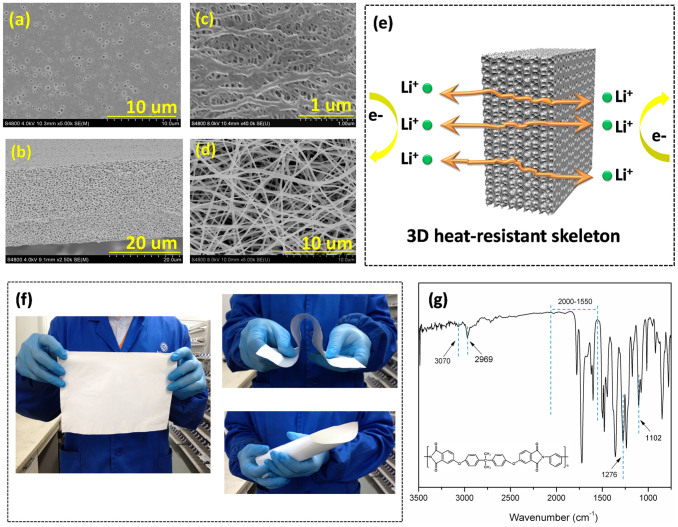
The SEM morphologies of (a), (b) the PEI based membrane, (c) The commercial PE separator and (d) the commercial nonwoven separator.(e) The schematic diagram of the PEI based membrane. (f) The flexibility of the PEI based membrane: digital photos. (g) The FTIR-ATR spectra of the PEI based membrane.

Figure 1a and 1b give the surface and cross-section morphologies of the PEI based membrane. As expected, a typical highly symmetric sponge-like structure was formed and uniformly distributed throughout the membrane. The pore size ranges from 0.1 ~ 0.5 μm. And the porosity is determined to be 70% (Table 1). High porosity is deemed to be favorable for high electrolyte uptake and high ion conductivity^16^. The morphologies of the referenced commercialized PE separator and heat-resistant polyimide (PI) non-woven separator are shown in Figure 1c and 1d. The PE separator is also prepared by phase inversion process with porosity of 38%. The PI separator is fabricated via the electrospining process and has a reticulated pore structure. The pore size is 5 μm and porosity is reported to be 80%.

To quantitatively illustrate the pore structure, Gurley value is evaluated and given in Table 1, which is determined by measuring the time for a settled volume (30 ml) of air to pass through the membrane with a fixed area (0.79 cm^2^) under the pressure of 0.02 MPa. Generally, high Gurley value corresponds to low air permeability and a long tortuous path for air transportation, implying higher curvature for pores^17^. The Gurley value of the PEI based separator is 217 sec 100 ml^−1^, higher than that of the referenced polyethylene (PE) separator (132 sec 100 ml^−1^). This kind of pore structure is believed to own high curvature to provide effective internal short circuit protection and to alleviate liquid electrolyte leakage and the occurrence of self-discharge^18^, which will be illustrated in the following sections in detail. Due to the large pore size and high porosity, the Gurley value of the PI nonwoven separator is beyond measuring limit, implying a straight-through pore structure. Figure 1e gives the schematic diagram of the PEI base 3D porous skeleton. The high curvature pore structure is believed to transport lithium ions and at the same time to help the battery to effectively avoid internal short circuit.

Figure 1f is the photograph of the prepared PEI based skeleton/membrane. As can be seen, through a simple and low-cost phase separation process, large area, flat, uniform and flexible porous membrane could be successfully achieved. Meanwhile, PEI base separator shows enough mechanical strength for practical use in lithium ion batteries (see Supplementary Figure S1 and Supplementary Table S1). These results suggest that the PEI based membrane is conducive to large-scale production and has high potential in practical applications.

The chemical compositions of the PEI based membrane are primarily confirmed by the ATR-FTIR spectrum (Figure 1g). The absorbance peak located at 3070 cm^−1^ corresponds to the stretching vibration of the = C-H bond. The stretching vibration of the saturated C-H bond appears at 2969 cm^−1^. The absorbance at 2000–1550 cm^−1^ represents the characteristic peaks of the benzene and -C = O bond. The stretching of the C-O bond and C-O-C bond in PEI chains creates the absorption at 1276 cm^−1^ and 1102 cm^−1^, respectively.

**Figure 2:**
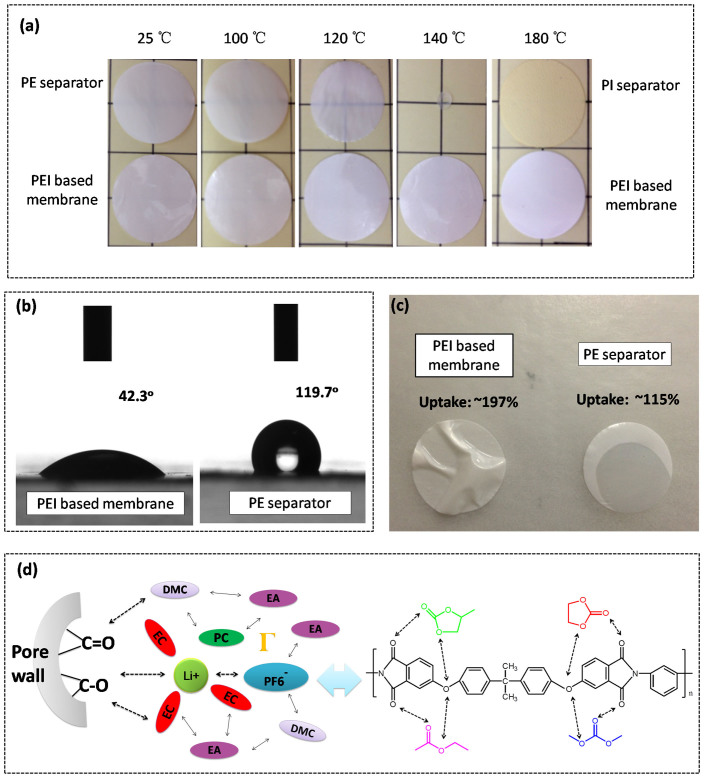
(a) The thermal shrinkage of the PEI based membrane, PE separator and PI nonwoven separator after being treated at different temperature for 1 h. (b) The contact angles of the PEI based membrane and the commercial PE separator with water. (c) The photograph of the wetting behavior of the separator with liquid electrolyte. (d) The schematic diagram of the interaction between PEI matrix and the liquid electrolyte.

Thermal stability is one of the most important technical parameters for separators, which must be considered when designing lithium ion batteries and choosing separators, especially for high power/energy ones. So far, to enhance battery safety, developing new kinds of separators with superior hest-resistance has become an important research direction^19^,^20^. The thermal stability of PEI based membrane is evaluated and compared with PE separator and PI separator. Samples were deposited in the oven at different temperatures for 1 h and the dimensional change was recorded. As shown in Figure 2a, PE separator shrinks completely when the temperature is raised to 140°C. The reason lies in that the melting point of PE is about 135°C. So PE separator totally melted down at 140°C. However, due to the relatively high heat deflection temperature (210°C, 0.45 MPa), the size of PEI based membrane shows no obvious change throughout the testing process. Meanwhile, the thermal stability of the PEI based membrane at 180°C weighs against that of the PI separator. This confirms the superior heat resistance of the PEI base membrane and the successful realization of the concept of the 3D heat-resistant skeleton, implying a promising contribution to enhance the thermal safety of lithium ion batteries when used as the separator^21^.

Owning enhanced compatibility with liquid electrolyte and achieving fast and uniform wetting by liquid electrolyte have long been an essential requirements for separators, especially for the ones used in the electric vehicles and energy storage systems, which necessitate large-sized cells. For polyolefin separators, the intrinsically low surface energy and hydrophobicity induce their poor wettability with high polar liquid electrolyte, which would bring additional disadvantages in manufacturing costs and cell performances^2^. However, due to the existence of the polar groups in PEI chains, the PEI based membrane shows higher surface energy (57.9 N m^−1^, Table 1) and better hydrophilicity (42.4°, Figure 2b) than the commercial PE separator (32.2 N m^−1^, Table 1 and 119.7°, Figure 2b), which indicates improved wettability. Xia et al^22^ have reported that the water contact angle of the Celgard polypropylene (PP) separator, the PET nonwoven separator and the polyvinyl alcohol (PVA)-*co*-PE composited PET nonwoven separator was 118.4°, 113.2° and 57.5°, respectively. By comparison, the PEI based membrane owns better hydrophilicity and wettability with high polar solvent than the previously reported separators.

The wetting ability of liquid electrolyte was further confirmed by adding a drop of liquid electrolyte on the surface of the separators. As can be seen in Figure 2c, the PEI based membrane is quickly wetted and on behalf of the high porosity, higher electrolyte uptake (197%, Table 1) is obtained. As depicted in Figure 2d, the compatibility of the PEI based matrix with the high polar solvent is effectively enhanced due to the strong interaction between the polar groups^23^. Enhanced affinity of PEI based separator with the liquid electrolyte is expected to contribute to better cyclability. Besides, higher uptake amount is expected to yield higher ion conductivity^24^,^25^. Thus, the ion conductivity at 25°C is determined and given in Table 1. The ambient ion conductivity of PEI based separator is 8.8 × 10^−4^ S cm^−1^, which is much higher than that of PE separator (1.2 × 10^−4^ S cm^−1^), which will be beneficial for improving the power performances in cell operations^26^.

## Discussion

**Figure 3:**
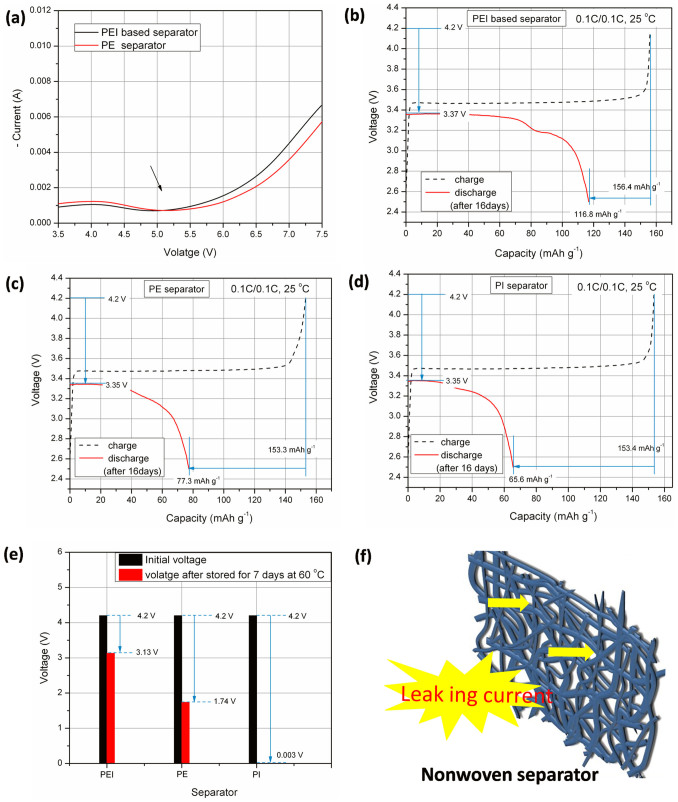
(a) The electrochemical stability window of the PEI based separator and the commercial PE separator (scan rate, 1 V s^−1^). The discharge capacity of cells containing (b) PEI based separator, (c) PE separator separator and (d) PI separator after the cells being deposited at 25°C for 16 days. (e) Comparison of the OCV drop of the cells containing PEI, PE and PI separators after being stored at 60°C for 7 days. (f) The schematic diagram of the nonwoven separator.

Since battery performances are going to be evaluated in this section, the PEI based skeleton/membrane will be coded as the PEI based separator in the following discussions. As a preliminary step to evaluate the battery performances of the separators, the electrochemical stability was firstly determined using the linear sweep voltammetry method. As plotted in Figure 3a, the electrochemical stability window of PEI based separator is higher than 5 V (vs. Li^+^/Li) and is comparable with that of the commercial PE one, suggesting that the PEI based separator is applicable for lithium ion batteries.

Cell performances of prepared separators were further evaluated by coin cells assembled with LiFePO_4_ cathode/separator/lithium anode. Firstly, the self-discharge properties of the cells were determined by evaluating the open-circuit voltage (OCV) drop and capacity decay, which could in turn predict internal short-circuits between electrodes^3^. The determination process includes charging cells to 4.2 V, recording the charge capacity and then after storing cells at 25°C and 60°C for 16 days and 7 days, respectively, cells being discharged, the initial OCV and discharge capacity being recorded. The results are given in Figure 3b, 3c, 3d and 3e. Cells assembled with PEI base separator show the lowest OCV drop (from 4.2 V to 3.37 V, Figure 3b) and discharge capacity decay rate (from 156.4 mAh g^−1^ to 116.8 mAh g^−1^, Figure 3b) after being stored for 16 days at 25°C. While the PI separator shows the most obvious self-discharge phenomenon (Figure 3d). Furthermore, the discrepancy of the cells containing the three kinds of separators becomes more obvious when cells were stored at 60°C for 7 days. The OCV of the PI separator decreases to 0.003 V from 4.2 V after the treatment. Due to the fact that OCV had dropped too much (below 2.5 V), discharge capacity was not determined. While, for PEI separator, the OCV drop is only 1.07 V, which is lower than that for PE and PI separators. The self-discharge behavior is known to be strongly dependent on the pore structure of the separators^27^. As depicted in Figure 3f, the large pore size and straight-through pores of the PI nonwoven separator easily cause internal short circuit, inducing OCV drop and capacity decrease. While, due to the high curvature of the pores of the PEI based separator (Figure 1e), the self-discharge phenomenon is effectively hindered. Results clearly show the advantage of PEI based separator over the PI nonwoven separator, although similar thermal stability could be achieved for these two separators.

**Figure 4:**
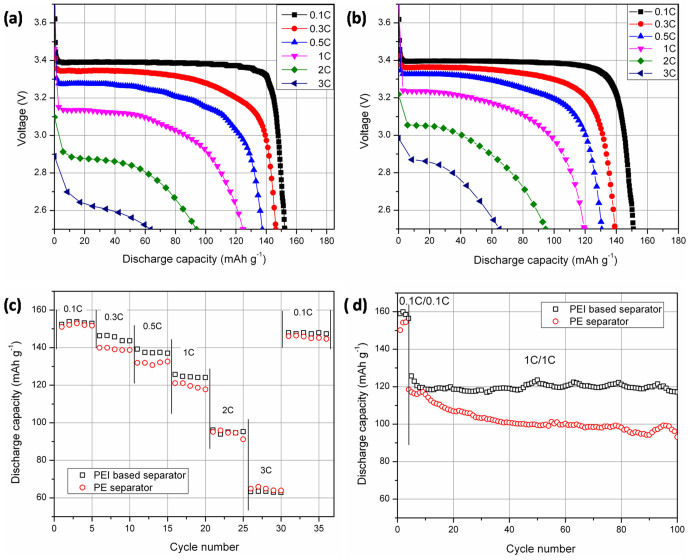
(a) The *C*-rate capacity of cells, which was assembled with LiFePO_4_ cathode and lithium anode, containing the PEI based separator. (b) The C-rate capacity of cells containing the commercial PE separator. (c) The comparison of the discharged C-rate capacity (the charge current is 0.1 C). (d) The discharge capacity of cells containing the PEI based separator and PE separator as a function of cycle number.

Figure 4 depicts the discharge capacity of the cells at various C-rates. Due to the straight pore structure being easy to cause internal short circuit (Figure 3f), the charge/discharge process of the coin cells containing PI separator could not proceed successfully. As shown in Figure 4a and 4b, the initial discharge capacity of the cells containing PEI based separator is 152.3, 146.4, 137.4, 124.7, 93.9 and 63.5 mAh g^−1^ at 0.1, 0.3, 0.5, 1, 2, 3 C rate, respectively. And that of the cells with PE separator is 151.0, 139.1, 130.7, 119.6, 94.6 and 64.9 mAh g^−1^ at 0.1, 0.3, 0.5, 1, 2, 3 C rate, respectively. By comparison, the C-rate capacity is almost equal. Figure 4c gives the summary of the rate capabilities. Due to higher ion conductivity, the discharge capacity of the cells containing PEI base separator is a little higher, when the discharge current rate is below 2 C. Decrease of the discharge capacity at higher current rate is attributed to the relatively lower surface porosity for the PEI based separator. Thus, to further improve the rate capacity, regulation and control of the surface pore structure would be the next diligent direction. Uniaxial or biaxial stretching might be a promising attempt to overcome this limitation.

The cycling performances of the cells at a constant charge/discharge current rate (1 C/1 C) were characterized as well (cycles at 0.1 C/0.1 C being the stable process). Figure 4d shows that the initial discharge capacity of cells containing the PEI based separator (125.7 mAh g^−1^) is higher than that of cells prepared with PE separator (118.5 mAh g^−1^). The discrepancy is attributed to higher ion conductivity for PEI based separator, which facilitates the repeated lithium ion intercalation/de-intercalation in/from the electrodes at relatively higher current rate (1 C), resulting in higher battery capacity^28^. The discharge capacity of the cells containing the PEI based separator is also compared with that of the previously reported separators. In Zhu et al's work^4^, the reversible discharge capacity of the polyvinylidene fluoride (PVDF)/nonwoven composite separator in the LiFePO_4_/lithium half cells was 120 mAh g^−1^ at 0.2 C. And the discharge capacity of the commercial PP separator (Celgard 2730) was 110 mAh g^−1^ at 0.2 C. The charging current rate was reported to be 0.2 C. While the stabled discharge capacity of the PEI based separator is about 120 mAh g^−1^ at 1 C with charging current rate of 1 C. Through contrast analysis, the coin cells containing the PEI based separator show higher discharge capacity than the previous result and than that of the commercial PP one.

The discharge capacity of the cells containing PE separator falls sharply during the first 100 cycles. The retention ratio of the discharge capacity is only 78.5%, while cells containing the PEI based separator show higher and more stable discharge capacity. The capacity retention ratio after 100 cycles is 93.7%. Improved cyclability is possibly due to stronger affinity between PEI based separator and liquid electrolyte, which contributes to better electrolyte retention during cycling and yields a higher capacity retention rate^29^.

**Figure 5:**
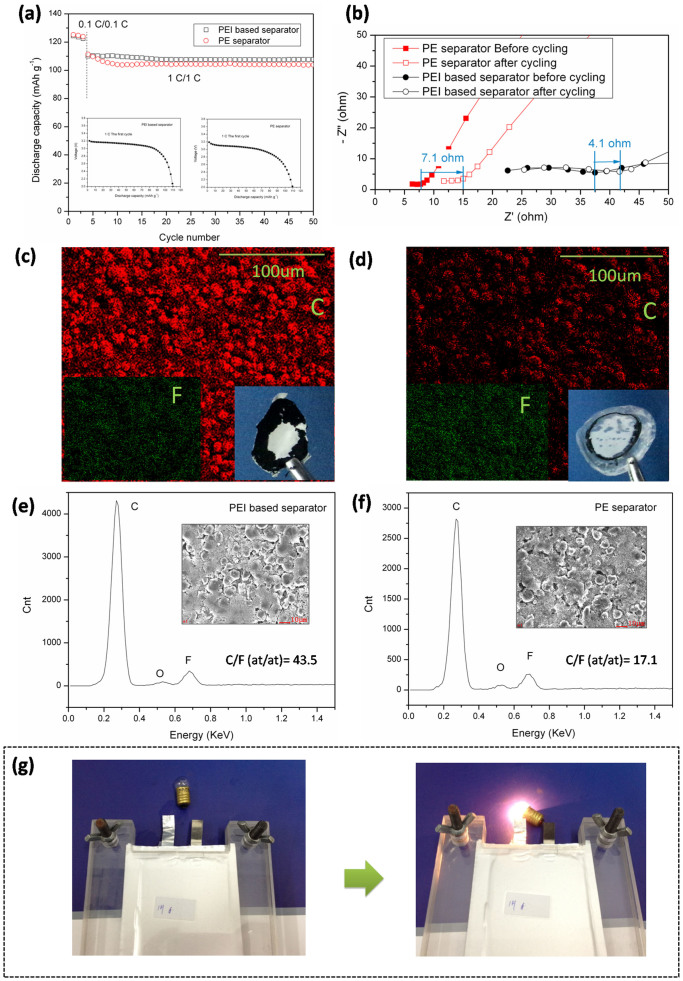
(a) Cell performances of the full cells with high-mass-loading electrodes (LiFePO_4_/separator/graphite). (b) Nyquist plots for the full cells measured before and after the 50 cycles. (c)–(f) Structural analysis of the anode surface after the cycling process, EDS analysis illustrating the distribution and concentration of C and F elements of (c), (e) PEI based separator and (d), (f) PE separator. The insets give the photograph of separator when the full cells were opened after the cycling process. (g) A demonstration of the LiMn_2_O_4_/graphite full cell operation. The charged cell could light up the 3.5 V small bulb.

The effect of the PEI base separator on battery performances was further investigated by analyzing cycle performances and the interfacial properties with the LiFePO_4_/separator-liquid electrolyte/graphite full cells under harsh operating conditions (i.e., the high-mass-loading electrodes and relatively fast charge/discharge rate). Figure 5a shows the discharge capacity of the full cells at 1 C as a function of the cycles. The LiFePO_4_ content in each full cell is about 17.7 mg. So the determined discharge capacity is relatively lower than that in the cells with low-mass-loading electrodes (Figure 4). The initial discharge capacity of the cells with PEI base separator at 1 C is 110.1 mAh g^−1^, while the initial discharge capacity at 1 C for the PE cells is 111.3 mAh g^−1^. However, the cells containing PE separator show a 93.3% capacity retention rate after 50 cycles. By comparison, cells containing PEI based separator exhibit more stable charge/discharge behavior (97.8% capacity retention rate) and smaller cell polarization.

To achieve better understanding of the improved cyclability of the PEI based cells. The interfacial stability of the full cells was evaluated by monitoring the variation of the impedance spectra during the cycling process. As shown in Figure 5b, the semicircle in the Nyquist plots represents the interfacial resistance^2^. The growth of the interfacial resistance (≈4.1 ohm) after the cycling process was suppressed when the PEI based separator was used in the full cells. Increase of the interfacial resistance is usually deemed as the formation of the undesirable resistive film^15^. The results suggest that PEI base separator is conducive to the interfacial stability of the cells. The reason is considered to be the superior wettability of the PEI separators with the liquid electrolyte, which could yield a more uniformly wetted and benign contact interfacial layer in the cells to restrict the growth of the undesirable resistive film^9^.

The interfacial properties were further illustrated by analyzing the surface compositions of the graphite anode with the EDS images. Compared with cells containing the PE separator, the full cells with the PEI base separator own a higher carbon concentration (labeled as red, Figure 5c and 5d) relative to the fluorine (labeled as green, Figure 5c and 5d). The concentration of the fluorine represents the content of the fluorine-containing byproducts, especially LiF byproducts, formed on the anode surface during the cycling process^15^. The quantitative value of C/F (at/at) was calculated and shown in Figure 5e and 5f. Same results were obtained that the carbon content is much higher relative to fluorine for cells containing the PEI base separator (C/F (at/at) = 43.5).

This fact further proves the unusual contribution of the PEI based separator to the cycling performances of the cells. The contribution could be explained as follows. First, as discussed above, the superior wettability of the PEI base separator allows for the uniform distribution of the liquid electrolyte and the compacted contact in the interfacial layer between the separator and the electrodes, which could restrict the growth of the byproducts (Figure 5b). The cells were opened and the adhesion condition between the separator and the anodes were observed. As shown in Figure 5c and 5d insets, the PEI based separator shows stronger adhesion to the anodes than the PE separator, which proves the above argument. Second, the high porosity and high ion conductivity give rise to favorable ionic transportation, which thus facilitates the Faradaic reaction of the electrodes and alleviate the cell polarization during the charge/discharge process^15^. According to Kim et al's opinions^15^, although the inert separators do not participate in the Faradaic reaction, the uniformly wetted electrode and more facial ionic transport could help to mitigating the unwanted side reaction between the liquid electrolyte and the electrode, which further contribute to better cell performances.

Finally, Figure 5f demonstrates that the charged (4.2 V) full battery containing the LiMn_2_O_4_ cathode, PEI based separator and the graphite anode could light up the 3.5 V small bulb, suggesting the decent operation of the battery system and the applicability of the PEI based separator in various battery systems^30^.

In summary, the PEI based 3D heat-resistant skeleton is successfully constructed and shows superior wettability with liquid electrolyte. The well-regulated 3D porous skeleton allows greatly improved ion transportation ability, at the same time provides a good short circuit protection function. The PEI based cells show good cycling performances and acceptable C-rate capacities. Considering the attractive features and the easily scale-up preparation process, the PEI based separator is deemed to have great promise for the application in high safety lithium ion batteries. A notable contribution of this study is providing a new type of separator for lithium ion batteries, which might be an interesting direction for the next-generation separator.

## Methods

### Sample collection

The PI nonwoven separator (thickness: 38 μm, porosity: 80%) was kindly supplied by Jiangxi Xiancai Science and Technology Co., Ltd., China. The commercial PE separator (thickness: 16 μm) was bought from Asahi Kasei Corp. The liquid electrolyte TC-E269 was purchased from Guangzhou Tinci Materials Technology Co., Ltd, China, which is 1.6 M LiPF_6_ in ethylene carbonate (EC)/propylene carbonate (PC)/dimethyl carbonate (DMC)/ethyl acetate (EA) with the mass ratio of 4:1:2:3 wt/wt/wt/wt. The liquid electrolyte S-3015A, which is 1 M LiPF_6_ in a mixture of ethylene and dimethyl carbonate (3:7 vol ratio), was provided by Zhangjiagang Guotai-Huarong New Chemical Materials Co., Ltd., China. Ultem PEI 1000 was purchased from General Electric Company. 1-Methyl-2-pyrrolidinone (NMP) was supplied by Shanghai Chemical Reagents Co., China.

### Separator preparation

PEI was dissolved in NMP to form a 20 wt.% transparent solution. The solution was then cast onto a clean glass plate with a thickness of 80 μm and quickly transferred to a constant climate chamber, kept at 50°C and 100% relative humidity. After 10 minutes, the sponge structured membrane was peeled off from the glass and thoroughly washed with water to remove the NMP. The resultant membrane was dried in the oven at 100°C for 4 h before use.

### Characterization

The morphologies of the separators and the EDS determination were characterized by field emission scanning electron microscopy (FESEM) (S-4800, Hitachi). The porosity was measured by immersing the separators in the isobutyl alcohol bath. After 2 h, the weight of saturated separators (*Ms*) was recorded. The porosity was determined according to the equation (1):
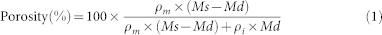
where *ρ_m_* and *ρ_i_* represent the density of the separator matrix and the isobutyl alcohol, respectively. *Ms* and *Md* represent the weight of the saturated separator and the dry separator, respectively. Gurley value was determined on a home-made instrument, which was defined as the time for a given amount of gas (30 ml) to pass through a fixed area (0.79 cm^2^) under 0.02 MPa pressure. The chemical composition of the PEI based separator was characterized by the attenuated total reflectance fourier transform infrared spectra (ATR-FTIR, Nicolet 6700, U.S.). The mechanical strength of separators was determined on a tensile tester (RGWT-4002, Shenzhen, China) at room temperature with a stretching rate of 200 mm min^−1^. The sample size is 30 mm × 10 mm. The contact angle and surface energy were measured by a contact angle measurement system (Dataphysics, OCA20, Germany) at 25°C. The electrolyte uptake (*U*) was achieved by determining the weight change of the separator before and after absorbing saturated liquid electrolyte, which was calculated by the equation (2):
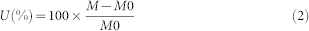
where *M0* and *M* represent the weight of the dry and wet separator, respectively.

The bulk impedance (*R_b_*) was achieved on an electrochemical work station system (CHI660e, China) with the assembly of stainless steel (SS)/separator/SS. The ion conductivity (*σ*) was calculated by the equation (3):
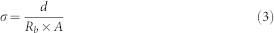
where *d* and *A* are the thickness and the effective area of the separator, respectively.

The rate capability and cycling stability were tested on LAND CT2001A system. The LiFePO_4_ half cells were cycled between 2.5 V and 4.2 V under different C-rate, in which the content of LiFePO_4_ in each cell is about 5.0 mg. The full cells with high mass-loading electrodes (LiFePO_4_ content being about 17.7 mg) were cycled between 2.0 V to 3.65 V. The interfacial resistance was determined by electrochemical work station system (Solartron analytical, 1470E, Britain).

## Author Contributions

J.L.S., Y.G.X., X.F.L. and H.M.Z. devised the original concept, designed the experiments. Z.Z.Y. fabricated the separator. J.L.S. and H.S.H. optimized separator and performed the electrochemical experiments. J.L.S., Y.G.X., X.F.L., H.M.Z. and Z.P.L. analyzed the results. J.L.S., Y.G.X. and X.F.L. co-wrote the paper. All authors viewed the manuscript.

## Supplementary Material

Supplementary Information Supporting information-mechanical strength

## Figures and Tables

**Table 1:** Summary of the basic characterizations of the PEI based separator and the PE separator

Separator	Porosity [%]	Gurley value [sec 100 ml^−1^]	Water contact angle [°]	Surface energy [N m^−1^, 25°C]	Electrolyte uptake [%]	Ion conductivity [S cm^−1^, 25°C]
PEI	~70%	217	42.3	57.9	~197	8.8 × 10^−4^
PE	~38%	132	119.7	32.2	~115	1.2 × 10^−4^
