# Supplementary material for: Porous membrane with high curvature, three-dimensional heat-resistance skeleton: a new and practical separator candidate for high safety lithium ion battery
Source: Sci Rep. 2015 Feb 5;5:8255. doi: 10.1038/srep08255 (PMC4317688; doi:10.1038/srep08255)
Supplement: Supplementary Information — Supporting information-mechanical strength [file srep08255-s1.pdf]

Porous membrane with high curvature, three-dimensional heat-resistance skeleton: a new and practical separator candidate for high safety lithium ion battery

Junli Shi[1], Yonggao Xia*[1], Zhizhang Yuan[2], Huasheng Hu[1], Xianfeng Li*[2], Huamin Zhang*[2] & Zhaoping Liu*[1]

[1] Ningbo Institute of Materials Technology Engineering (NIMTE), Chinese Academy of Sciences, Zhejiang 315201, P. R. China, [2] Division of energy storage, Dalian Institute of Chemical Physics, Chinese Academy of Sciences, Dalian 116023, China

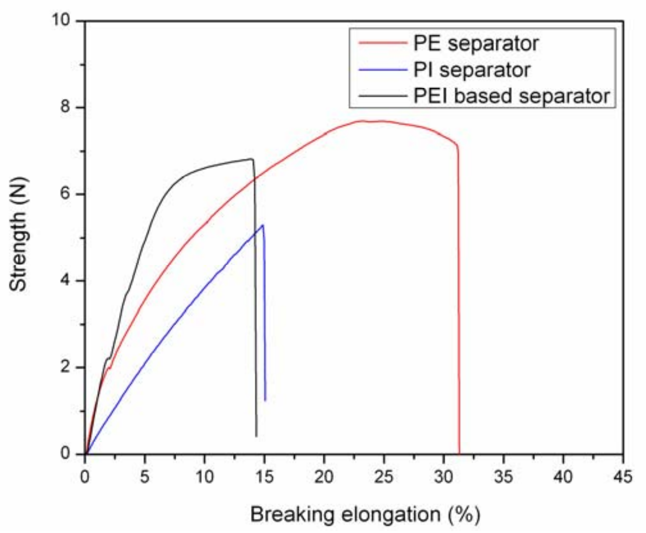

Supplementary Figure S1 The tensile curves of the PEI based separator, PE separator and PI separator.

Supplementary Table S1 Mechanical strength of the PEI based separator, PE separator and PI separator

Separator	Breaking elongation [%]	Tensile strength [MPa]
PEI based separator	14.3%	34.0
PE separator	31.3%	48.1
PI separator	15.1%	13.9

Discussion

The mechanical strength of the PEI based separator, PE separator and PI separator was characterized by the determining the tensile curves. The results could be seen in Figure S1 and Table S1. The breaking elongation of the PEI based separator and the PI

separator is comparable. But the tensile strength of the PEI based separator is obviously higher than that of the PI separator. Since the PI separator has been widely produced and widely used, it is deemed to own proper mechanical strength for the application in lithium ion batteries. Considering better mechanical strength for the PEI based separator, it is reasonable to conclude that the PEI base separator could satisfy the mechanical strength requirements for practical use in lithium ion batteries.

The commercial PE separator owns the highest elongation and tensile strength than the other two separators. Better mechanical strength comes from relatively higher crystallinity and the orientation of polymer segments driven by the biaxial stretching process in separator prepared process. [1, 2] Although the mechanical strength of the PEI based separator is relatively lower than that of the PE one. According to previous reports, [1, 2] the after-stretching process combining with the heat treatment process could further improve the mechanical properties of the PEI base separator, which is also the direction of our future work.

1 Gao, K., Hu, X. G., Dai, C. S. & Yi, T. F. Crystal structures of electrospun PVDF membranes and its separator application for rechargeable lithium metal cells. *Mater. Sci. Eng. B* **131**, 100–105 (2006).

2 Liang, Y. Z. et al. Heat treatment of electrospun Polyvinylidene fluoride fibrous membrane separators for rechargeable lithium-ion batteries. *J. Power Sources* **240**, 204–211 (2013).
